# The efficacy and safety of Xuefu Zhuyu Decoction combined Mifepristone in the treatment of Uterine leiomyoma: A protocol systematic review and meta-analysis

**DOI:** 10.1097/MD.0000000000024306

**Published:** 2021-01-22

**Authors:** Shasha Shi, Qiaobo Ye, Chenghao Yu, Fu Peng

**Affiliations:** a Department of Basic Medicine, College of Chengdu University of Traditional Chinese Medicine; b Department of West China School of Pharmacy, College of Sichuan University, Chengdu, Sichuan, China.

**Keywords:** meta-analysis, randomized controlled trial, uterine leiomyoma, xuefu zhuyu decoction

## Abstract

**Background:**

Uterine leiomyoma (UL) is a common severe gynecological issue. In China, Xuefu Zhuyu Decoction (XFZYD), combined with Mifepristone, is widely used in the treatment of UL. However, their combined effectiveness and safety for this purpose have not yet been explored.

**Objective:**

This systematic review aims to evaluate the effectiveness and safety of XFZYD combined with Mifepristone as a method of treatment for UL.

**Methods:**

We searched the following 7 databases: 3 English medical databases (PubMed, EMBASE, Cochrane Library), and 4 Chinese medical databases (Chinese Biomedical Literature Database (CBM), Chinese National Knowledge Infrastructure (CNKI), Chinese Scientific Journal Database (VIP), and the Wanfang database). The primary outcome was the effect of XFZYD combined with Mifepristone on the effective rate, uterine leiomyoma volume (ULV), and uterine volume (UV) of uterine leiomyoma. Bias risk was assessed using the Cochrane risk of bias tool. The software RevMan5 was used to evaluate the quality of the included studies and process the data.

**Results:**

This study will evaluate the efficacy and safety of XFZYD combined with Mifepristone in the treatment of uterine fibroids by evaluating the effective rate, Uterine Leiomyoma volume, and uterine volume, the incidence of estradiol, luteinizing hormone, and other indicators.

**Conclusion:**

This study will provide reliable evidence-based evidence for Xuefu Zhuyu Decoction Combined with Mifepristone in the treatment of uterine fibroids.

**Ethics and dissemination:**

Private information from individuals will not be published. This systematic review also does not involve endangering participant rights. Ethical approval will not be required. The results may be published in a peer-reviewed journal or disseminated at relevant conferences.

**OSF Registration number:**

DOI 10.17605/OSF.IO/YADN3

## 1 Introduction

Uterine leiomyoma (UL), also known as fibroma or leiomyoma, is a female gynecologic tumor^[1]^ with an incidence of about 20% to 77%.^[2]^ It is closely related to increasing age, weight, and waist circumference and can be controlled by surgery or drug therapy.^[3,4]^ Although the surgery is minimally invasive,^[5]^ it causes women immeasurable pain and increases medical expenses.^[6]^ Therefore, the nonsurgical treatment of UL is of particular relevance. At present, conventional drugs used for treating UL include GnRH agonists, aromatase inhibitors, and dopamine receptor agonists, among others.^[7]^

Traditional Chinese medicine (TCM) has been used in the treatment of diseases for thousands of years and has gained the overwhelming trust of patients through its history of positive therapeutic effects. Xuefu Zhuyu Decoction (XFZYD) is composed of the peach kernel (Tao Ren), safflower (Hong Hua), angelica (Dang Gui), radix rehmanniae (Sheng Di), chuanxiong rhizome, red peony root (Chi Shao), achyranthes root (Niu Xi), balloonflower (Jie Geng), Chinese thorowax (Chai Hu), bitter orange (Zhi Ke), and glycyrrhiza (Gan Cao).^[8]^ Clinical studies have confirmed that Xuefu Zhuyu Decoction has a good effect on diseases of nervous system, digestive system, cardiovascular system, and obstetrics and gynecology.^[9]^ It can treat gynecological diseases such as chronic pelvic inflammation,^[10]^ irregular menstruation,^[11]^ and ovarian cysts^[12]^ caused by blood stasis. Research on the modern mechanism of XFZYD has shown that the formula can play a therapeutic role by regulating hormone levels in the treatment of UL.^[13]^

Some clinical studies published in China have shown that XFZYD is often used in combination with Mifepristone in UL treatment with satisfactory therapeutic effects,^[14–16]^ but there is no systematic evidence to support these findings. Therefore, this systematic review and meta-analysis is the first comprehensive evaluation of the efficacy and safety of XFZYD combined with Mifepristone in the treatment of UL, to provide evidence for clinical use.

## 2 Methods

### 2.1 Protocol register

This protocol of systematic review and meta-analysis has been drafted under the guidance of the preferred reporting items for systematic reviews and meta-analyses protocols (PRISMA-P). Moreover, it has been registered on open science framework (OSF) (Registration number: DOI 10.17605/OSF.IO/YADN3).

### 2.2 Ethics

Ethical approval is not required for this study since it relies on secondary data.

### 2.3 Inclusion and exclusion criteria

The inclusion criteria of our review specified that: all participants in the included studies were patients with confirmed UL, without any restrictions concerning age and race; the treatment method in the intervention group was XFZYD combined with Mifepristone, while the treatment method in the control group was Mifepristone alone; all included studies were RCTs; and studies were from articles published in Chinese or English.

Exclusion criteria ruled out any studies that: displayed a lack of literature on related outcome indicators; had been selected for duplicate published studies; was impossible to extract the related data from the published results, and unable to obtain the primary data after contacting the author; the treatment group adopted other traditional Chinese medicine therapies, such as acupuncture and moxibustion, or other traditional Chinese medicine, the control group was not treated with Mifepristone alone.

### 2.4 Outcome measures

The primary outcomes included the effective rate, uterine leiomyoma volume (ULV, cm^3^), and uterine volume (UV, cm^3^). The secondary outcomes included the total incidence of adverse events (%), the incidence of estradiol (E2, pmol/L), luteinizing hormone (LH, IU/L), follicle-stimulating hormone (FSH, IU/L), and progesterone (P, nmol/L).

### 2.5 Search strategy

We searched for randomized controlled trials (RCTs) reported through December 2020; the electronic databases included: the Cochrane Library, PubMed, Embase, Chinese Biomedical Literature Database (CBM), Chinese National Knowledge Infrastructure (CNKI), Chinese Scientific Journal Database (VIP), and the Wanfang database. As a herbal formula of traditional Chinese medicine, XFZYD is mainly used in China, so the 4 electronic databases of China were searched to obtain a comprehensive study.

The keywords used, respectively, or in combination, to retrieve relevant published studies from 7 electronic databases included: “Xuefu Zhuyu Decoction,” “Xuefu Zhuyu,” “uterine leiomyoma,” “uterine fibroids,” and so on. This retrieval strategy was determined after multiple searches. PubMed retrieval strategies are shown in Table 1. The aim was to locate RCTs of XFZYD in combination with Mifepristone for the treatment of UL, filter documents, and extract data according to inclusion and exclusion criteria.

**Table 1:** Search strategy in PubMed database.

Number	Search terms
#1	Uterine leiomyoma [MeSH]
#2	Uterine fibroids [Title/Abstract]
#3	Fibroma, Uterine [Title/Abstract]
#4	Fibroid, Uterine [Title/Abstract]
#5	Leiomyoma, Uterine [Title/Abstract]
#6	Tumors, Fibroid [Title/Abstract]
#7	Fibroid Uterus [Title/Abstract]
#8	#1 OR #2 OR #3 OR #4 OR #5 OR #6 OR #7
#9	Xuefu Zhuyu [Title/Abstract]
#10	XFZY [Title/Abstract]
#11	#9 OR #10
#12	#8 And #11

### 2.6 Study selection and data extraction

**Figure 1:**
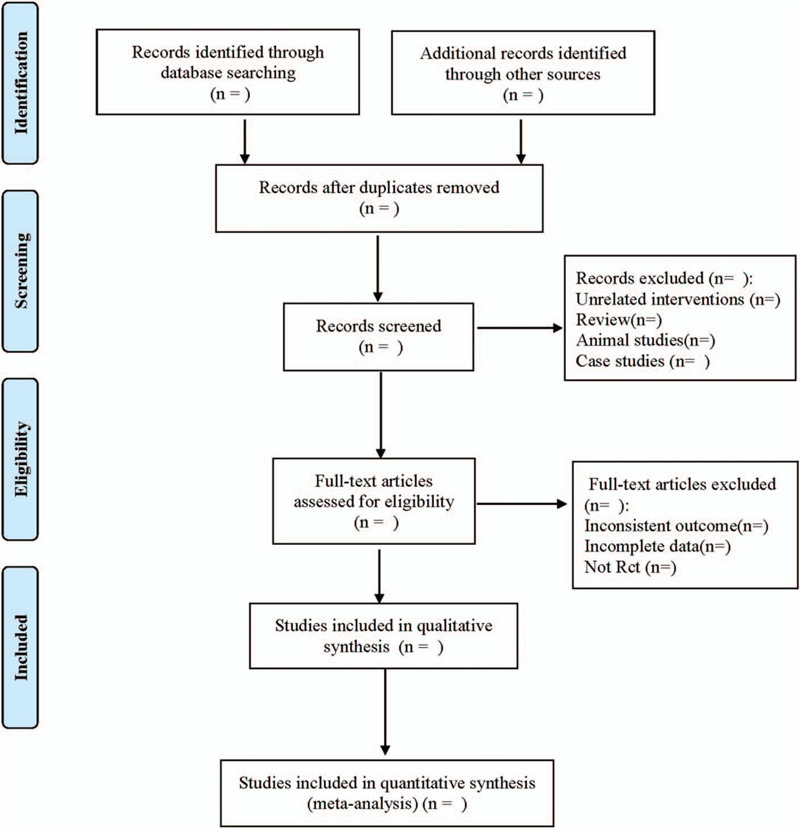
Flow diagram.

The 2 reviewers (SS, QY) read the title and abstract of the article independently, and then read the full text after screening to determine whether the final article should be included. Any differences were resolved by consensus or in consultation with a third party (FP). The integration process was shown as a flow diagram (Fig. 1). The data was extracted and managed by the 2 reviewers. The data collection form was designed in advance, and each reviewer independently extracted and collected data from the articles involved, including general trial characteristics (title, author, year); baseline data (sample size, age, sex, disease course); intervention measures (Chinese patent medicine dose, control intervention medication, intervention time); results (primary outcome indicators, secondary outcome indicators, adverse events). If there was a difference in perspective between the 2 reviewers, it was settled through negotiation or with assistance from a third party (FP).

### 2.7 Quality assessment

The 2 reviewers assessed the quality of the included study using the risk of bias tool recommended by the Cochrane collaboration. The risk of bias assessment was divided into 6 items, according to the Cochrane Handbook: random sequence generation; concealed allocation; blind implementation of participants and personnel; blind method of outcome assessment; incomplete result data; selective reporting, and other sources of bias. Each project had 3 categories: “high risk,” “low risk,” and “unclear.” Other deviations include profit deviations and sample calculations. In the case of inconsistency, the 2 reviewers negotiated between themselves or with a third party to resolve the difference.

### 2.8 Statistical analysis

This systematic review used Review Manager RevMan 5.3(developed by the UK's International Cochrane Collaboration) software (to analyze the data and *I*^*2*^ statistics to evaluate the statistical heterogeneity of the research results. If *I*^*2*^* *< 50%, the heterogeneity between the 2 was considered acceptable, and a fixed-effect model was adopted. If *I*^*2*^ > 50%, it was considered that there was significant heterogeneity; the source of heterogeneity was then explored through subgroup analysis or sensitivity analysis. If there was no apparent clinical or methodological heterogeneity, it was considered statistical heterogeneity, and the random-effect model was used for analysis. Descriptive analysis was used if there was significant clinical heterogeneity between the 2 groups and subgroup analysis was not available. Risk ratio was used for binary results, and weighted mean differences (WMDs) were used for continuous results. The WMD was used for continuous variables with the same units of weights and measures, and standardized (S)MD was used for continuous variables with different units of weights and measures and large differences in meaning. For all the estimates, we calculated the 95% CI.

### 2.9 Subgroup and sensitivity analyses

Subgroup analysis focused on the effect of different intervention characteristics on the cure rate, such as the duration of treatment, variance in the dosage of agents, and so on. A sensitivity analysis was performed to verify the stability of the results. After each document was excluded, the new results were analyzed again and compared with the original consolidated results to see if the study had affected the stability of the results.

### 2.10 Assessment of reporting bias

For the significant outcome indicators, a funnel plot was used to qualitatively detect publication bias when the number of the included study was ≥10. Egger and Begg tests were used to assess potential publication bias quantitatively.

### 2.11 Evidence quality evaluation

We grade the outcome indicators through the Grading of Recommendation Assessment, Development and Evaluation (GRADE) method.^[17]^ The evaluation includes bias risk, indirectness, inconsistency, inaccuracy, and publication bias. The quality of evidence will be rated as high, medium, low, or very low.

## 3 Discussion

As the incidence of UL increases, the cost of treatment plays a vital role in the world's health and economic burden.^[18]^ Therefore, the development of effective drugs or methods for the treatment of UL is incredibly important. At present, TCM and complementary alternative treatments for UL are garnering increasing attention. In China, TCM has a long history of use as adjuvant therapy for UL and is recognized by clinicians. Some studies have pointed out that TCM combined with Western medicine to treat UL can reduce the dosage of Western medicine, reduce the cost, and is expected to become a substitute for Western medicine.^[19]^ The ingredients in TCM formulae are diverse and effective and can be used to treat UL in a multitargeted and multi-pathway manner.^[20]^

The TCM compound XFZYD is one of the decoctions commonly used in the clinical treatment of UL. The formula has the therapeutic properties of promoting blood circulation, removing blood stasis, and alleviating pain; these functions have been widely recognized in China.^[21]^ Modern studies have confirmed that salvia miltiorrhiza, the main ingredient in Xuefu Zhuyu Decoction, has obvious inhibitory effect on the growth and proliferation of uterine fibroids;^[22]^ The total saponins of Panax notoginseng can improve platelet aggregation and adhesion, reduce blood viscosity, and have antitumor effect.^[23]^ The active components of safflower can inhibit tumor growth by inhibiting cell growth factor and blocking cell transfer pathway.^[24]^

Although a number of clinical studies have confirmed the efficacy and safety of Xuefu Zhuyu Decoction combined with mifepristone in the treatment of uterine fibroids, the methods and results of various studies are different. Therefore, based on the current evidence, this study will evaluate the effectiveness of Xuefu Zhuyu Decoction combined with mifepristone in the treatment of uterine fibroids, providing a clinical basis for the clinical application of this treatment regimen.

However, this system evaluation has some limitations. There may be some clinical heterogeneity due to different drug doses, dosage forms, and patients’ disease degrees among the included studies. Due to the different follow-up time of different studies, there are differences in the evaluation of long-term efficacy and safety. Due to the limitation of language retrieval, we will only include Chinese and English literature, and may ignore studies in other languages and regions.

## Author contributions

**Administrative support:** Shasha Shi.

**Collection and assembly of data:** Shasha Shi, Qiaobo Ye.

**Conception and design:** Chenghao Yu.

**Conceptualization:** Chenghao Yu.

**Data analysis and interpretation:** Shasha Shi, Qiaobo Ye.

**Data curation:** Shasha Shi, Qiaobo Ye.

**Final approval of manuscript:** Shasha Shi, Qiaobo Ye, Chenghao Yu, Fu Peng.

**Manuscript writing:** Shasha Shi, Fu Peng.

**Provision of study materials or patients:** None.

**Resources:** Qiaobo Ye.

**Writing – original draft:** Shasha Shi, Fu Peng.

**Writing – review & editing:** Shasha Shi, Qiaobo Ye, Fu Peng.

## References

[1] Borahay MA Asoglu MR Mas A Estrogen receptors and signaling in fibroids: role in pathobiology and therapeutic implications. Reprod Sci 2017;24:1235–44. 27872195 10.1177/1933719116678686 PMC6344829

[2] Baird DD Dunson DB Hill MC High cumulative incidence of uterine leiomyoma in black and white women: ultrasound evidence. Am J Obstet Gynecol 2003;188:100–7. 12548202 10.1067/mob.2003.99

[3] Niles KM Blaser S Shannon P Fetal arthrogryposis multiplex congenita/fetal akinesia deformation sequence (FADS)—aetiology, diagnosis, and management. Prenatal Diagn 2019;39:720–31. 10.1002/pd.5505 31218730

[4] Sharami SH Fallah Arzpeyma S Shakiba M Relationship of uterine fibroids with lipid profile, anthropometric characteristics, subcutaneous and preperitoneal fat thickness. Arch Iran Med 2019;22:716–21. 31823623

[5] Marín-Buck A Karaman E Amer-Cuenca JJ Minimally invasive myomectomy: an overview on the surgical approaches and a comparison with mini-laparotomy. J Invest Surg 2019 1–8. 10.1080/08941939.2019.1642422 31322011

[6] Fernandez H Jourdain O Villefranque V Economic impact of ulipristal acetate on surgical procedures for uterine fibroids in France. BMJ Open 2017;7:e015571. 10.1136/bmjopen-2016-015571 PMC5588963 28871011

[7] Ciebiera M Łukaszuk K Męczekalski B Alternative oral agents in prophylaxis and therapy of uterine fibroids-an up-to-date review. Int J Mol Sci 2017;18:2586. 10.3390/ijms18122586 PMC5751189 29194370

[8] Qingren W Traditional Chinese Medicine Clinical Practical Classic Series Yilingaicuo Large Edition. Beijing: China Medical Science and Technology Press; 2018.

[9] Fu C Xia Z Liu Y Qualitative analysis of major constituents from Xue Fu Zhu Yu Decoction using ultra high performance liquid chromatography with hybrid ion trap time-of-flight mass spectrometry. J Sep Sci 2016;39:3457–68. 27384131 10.1002/jssc.201600083

[10] Xiuping JIN Yinglan MA Study on efficacy of modified Xuefu Zhuyu Decoction in chronic pelvic inflammation and part of its mechanisms. J World Chin Med 2020;15:421–5.

[11] Jun Z Clinical observation of 50 cases of Irregular menstruation treated by Xuefu Zhuyu Decoction. J Chin Pract Med 2008 87–8.

[12] Li P Mao DX Clinical observation of Guizhi Poring Pill combined with Xuefu Zhuyu Decoction in the treatment of ovarian cyst. J Basic Chin Med 2019;25:1155–6.

[13] Zhou L Li S Zhang E Effect of Xuefu Zhuyu Decoction on NOS, TNF- a and IL- 2 levels and uterine smooth muscle thickness in rats with uterine leiomyoma. J World Chin Med 2019;14:1408–11.

[14] Wang H Cheng W Study on the effect of Mifepristone combined with Xuefu Zhuyu Decoction in the treatment of uterine fibroids. J Contemporary Med Symp 2019;17:113–4.

[15] Yu J Wang X Clinical effect and safety of Mifepristone with Xuefu Zhuyu Jiaonang for uterine fibroids. J Liaoning J Traditional Chin Med 2015;42:128–30.

[16] Yu P-S Zhang TH Clinical observation of Xuefu Zhuyu Decoction combined with Mifepristone in the treatment of uterine fibroids. J Mod J Integr Traditional Chin Western Med 2014;23:1530–2.

[17] Puhan MA Schünemann HJ Murad MH A GRADE Working Group approach for rating the quality of treatment effect estimates from network meta-analysis. BMJ 2014;349:g5630. 25252733 10.1136/bmj.g5630

[18] Harrington A Bonine NG Banks E Direct costs incurred among women undergoing surgical procedures to treat uterine fibroids. J Manag Care Spec Pharm 2020;26: 1-a suppl: S2–10. 31958025 10.18553/jmcp.2020.26.1-a.s2 PMC10408391

[19] Su SY Muo CH Morisky DE Use of Chinese medicine correlates negatively with the consumption of conventional medicine and medical cost in patients with uterine fibroids: a population-based retrospective cohort study in Taiwan. BMC Complement Altern Med 2015;15:129. 25902837 10.1186/s12906-015-0645-0 PMC4414285

[20] Li R Li Q Ji Q Molecular targeted study in tumors: from western medicine to active ingredients of traditional Chinese medicine. Biomed Pharmacother 2020;121:109624. 31733579 10.1016/j.biopha.2019.109624

[21] Tang SQ Chen YH Chen XP In vivo effect of guiding-herb radix platycodonis and radix cyathulae on paeoniflorin pharmacokinetics of xuefu zhuyu tang in rats. Afr J Tradit Complement Altern Med 2017;14:289–96. 28638892 10.21010/ajtcam.v14i4.32 PMC5471477

[22] Chang YJ Lee DU Nam DY Inhibitory effect of Salvia plebeia leaf extract on ultraviolet-induced photoaging-associated ion channels and enzymes. Exp Ther Med 2017;13:567–75. 28352332 10.3892/etm.2017.4025 PMC5348704

[23] Han F-j Teng Y Wang X-x Advances of traditional Chinese medicine on hysteromyoma. J Guiding J Traditional Chin Med Pharm 2014;20:51–4.

[24] Xia J Miao M Modern research and new applications of safflower. J China J Chin Med 2013;28:1682–5.

